# Sequence-specific capture and concentration of viral RNA by type III CRISPR system enhances diagnostic

**DOI:** 10.21203/rs.3.rs-1466718/v1

**Published:** 2022-04-19

**Authors:** Anna Nemudraia, Artem Nemudryi, Murat Buyukyoruk, Andrew M. Scherffius, Trevor Zahl, Tanner Wiegand, Shishir Pandey, Joseph E. Nichols, Laina Hall, Aidan McVey, Helen H Lee, Royce A. Wilkinson, Laura R. Snyder, Joshua D. Jones, Kristin S. Koutmou, Andrew Santiago-Frangos, Blake Wiedenheft

**Affiliations:** 1 Department of Microbiology and Cell Biology, Montana State University, Bozeman, MT 59717, USA; 2 These authors contributed equally; 3 These authors contributed equally; 4 Department of Chemistry, University of Michigan, Ann Arbor, MI 48105, USA; 5 Lead contact

## Abstract

Type-III CRISPR-Cas systems have recently been adopted for sequence-specific detection of SARS-CoV-2. Here, we make two major advances that simultaneously limit sample handling and significantly enhance the sensitivity of SARS-CoV-2 RNA detection directly from patient samples. First, we repurpose the type III-A CRISPR complex from *Thermus thermophilus* (TtCsm) for programmable capture and concentration of specific RNAs from complex mixtures. The target bound TtCsm complex primarily generates two cyclic oligoadenylates (i.e., cA_3_ and cA_4_) that allosterically activate ancillary nucleases. To improve sensitivity of the diagnostic, we identify and test several ancillary nucleases (i.e., Can1, Can2, and NucC). We show that Can1 and Can2 are activated by both cA_3_ and cA_4,_ and that different activators trigger changes in the substrate specificity of these nucleases. Finally, we integrate the type III-A CRISPR RNA-guided capture technique with the Can2 nuclease for 90 fM (5×10^4^ copies/ul) detection of SARS-CoV-2 RNA directly from nasopharyngeal swab samples.

## Introduction

Although qPCR (quantitative polymerase chain reaction) remains the “gold standard” for nucleic acid detection, it requires sophisticated equipment, trained personnel, efficient specimen transport to high-complexity labs, and reliable reporting systems^1^. While the complexity and turnaround times necessary for qPCR are acceptable for many diagnostic applications, the SARS-CoV-2 (Severe Acute Respiratory Syndrome Coronavirus 2) pandemic reveals an urgent need for diagnostics that are easy to distribute, simple to perform, and fast enough to stop transmission of a contagious disease^1^. Although rapid antigen tests and isothermal amplification methods have helped address this need, these and other emerging methods have limitations related to sensitivity, versatility, or specificity^2,3^.

CRISPR RNA-guided diagnostics (CRISPR-dx) are a diverse group of nascent technologies that aim to address current limitations by providing a versatile and programmable platform that is sufficiently sensitive for clinical applications and stable enough for distribution^4,5^. The first CRISPR-based viral diagnostic came from Collins and colleagues in 2016, when they demonstrated that Cas9 could be used to discriminate between different variants of the Zika virus^6^. This approach relies on converting viral RNA to DNA using reverse transcriptase, followed by isothermal DNA amplification prior to sequence-based discrimination by Cas9. The exclusive recognition of double-stranded DNA (dsDNA) by Cas9 seemed to be an intrinsic limitation for diagnostic applications that require RNA detection. However, Beisel and colleagues recently developed a creative method that uses the trans-acting CRISPR-RNA (tracrRNA) to capture complementary RNA guides derived from RNA viruses^7^. In this system, the engineered tracrRNA-crRNA hybrid guides Cas9 to a complementary dsDNA reporter. While this approach enables RNA detection, Cas9 is a single turn-over enzyme, which may limit its sensitivity. In contrast to Cas9, target recognition by type V (Cas12-DETECTR) and type VI (Cas13-SHERLOCK) CRISPR-systems activates a multi-turnover non-sequence-specific “collateral nuclease” activity that amplifies the signal by cleaving thousands of reporter molecules for every target bound^8,9^.

Like type VI, type III systems also recognize complementary RNA. However, unlike any other CRISPR system, target recognition by type III complexes simultaneously activates polymerase and HD-nuclease domains in the Cas10 subunit^10–12^. The polymerase domain has been estimated to generate ~1000 cyclic oligoadenylates per bound RNA^13^, which trans-activate and allosterically regulate diverse multi-turnover ancillary nucleases that provide defense from invading genetic parasites^14,15^. This biochemical cascade exponentially amplifies the signal when a type III complex detects target RNA, suggesting that these systems have the potential to enhance the sensitivity of CRISPR-based diagnostics. However, initial efforts to implement this approach failed to be sufficiently sensitive for clinical applications without prior amplification of the target RNA^16–18^. The sensitivity of this first-generation diagnostic was in part limited by the use of Csm6 ancillary nucleases that also degrade the cyclic nucleotide activator^19–23^. Recently, Malcolm White’s lab demonstrated that alternative ancillary nucleases, which efficiently cleave reporters but do not cleave the signaling molecule, can be used to enhance the sensitivity of type III-based diagnostics^24^.

Despite innovations leading to new and improved CRISPR-based diagnostics, point-of-care testing requires new strategies that simplify the workflow and increase the sensitivity without prior RNA purification or amplification (e.g., PCR, LAMP, NASB, RPA, etc.). Here, we bring CRISPR-dx closer to a deployable diagnostic by developing a type III CRISPR-based method for sequence-specific capture and concentration of RNA from heterogeneous samples. To improve the sensitivity, we purify several different ancillary nucleases (i.e., Can1, Can2, and NucC), systemically test nuclease activation using a series of purified cyclic oligoadenylate standards (i.e., cA_3_-cA_6_), test for ring nuclease activity and determine how cyclic oligoadenylates, as well as metal-preferences impact substrate cleavage activities. We show that the Can1 nuclease from *T. thermophilus* (TtCan1) and the Can2 ortholog from *Archaeoglobi* archaeon JdFR-42 (AaCan2) are activated by more than one cyclic nucleotide species (i.e., cA_3_ and cA_4_) and that substrate specificity of these nucleases changes according to the bound activator. This observation helps to explain how diverse cyclic nucleotides (i.e., cA_3_-cA_6_) produced by a single type III surveillance complex integrate distinct activities from a single effector. Finally, we demonstrate how the type III complex can be used to bypass RNA extraction methods, and that coupling type III-based RNA capture with the AaCan2 nuclease further increases the sensitivity of SARS-CoV-2 RNA detection in patient swabs to 5×10^4^ copies/ul.

## Results

### Type III-mediated sequence-specific enrichment of RNA

**Fig. 1:**
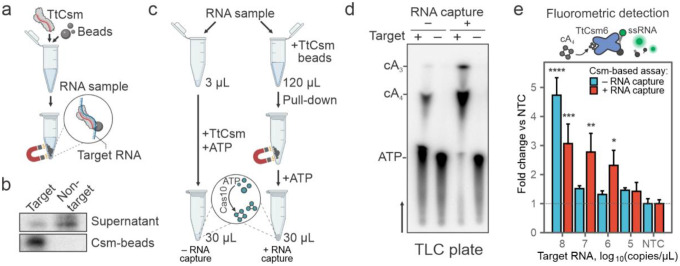
Type III CRISPR-based RNA concentration enhances detection. **a** Schematic of Type III CRISPR-based RNA concentration. RNase-dead Type III CRISPR complex from *Thermus thermophilus* (e.g., TtCsm^Csm3-D34A^) is added to a sample to bind complementary “Target” RNA. The His-tagged complex is concentrated using nickel-derivatized magnetic beads and a magnet. **b** Sequence-specific RNA enrichment with TtCsm^Csm3-D34A^ complex was tested using 25 nM ^32^P 5`-end labeled RNA. Target and non-target RNA fragments were mixed with 125 nM TtCsm^Csm3-D34A^ complex, incubated at 65°C for 20 min prior to concentration of the His-tagged complex with nickel-derivatized magnetic beads. After the pull-down, phenol-chloroform extracted RNAs from the supernatants and the Csm-beads were resolved using UREA-PAGE. **c** Csm-based direct RNA detection using 3 μL of sample is compared to an assay with an additional RNA capture and concentration step. Magnetic beads decorated with TtCsm^Csm3-D34A^ are added to the sample. After concentrating beads with a magnet, the supernatant is decanted. The pellet is then resuspended in a small volume of the reaction buffer containing ATP to activate polymerase activity of Cas10. Polymerization products (e.g., cA_3_ and cA_4_) are used for the downstream detection assays. **d** TtCsm^Csm3-D34A^ polymerization reactions were performed with α−^32^P-ATP as shown in **c** and products were resolved using thin-layer chromatography (TLC). Black arrow shows migration of solvent in the TLC plate. Bands were annotated using chemically synthesized standards (Supplementary Fig. 1d). 3 μL (− RNA capture) or 120 μL (+ RNA capture) of SARS-CoV-2 N-gene RNA (10^10^ copies/μL) diluted in total human RNA (293T cells) were used for reactions. **e** TtCsm6-based fluorescent readout (top panel) is used for detection of cA_4_ generated by TtCsm^Csm3-D34A^ with (red bars) or without RNA capture step (blue bars) as shown in panel **c**. SARS-CoV-2 N-gene RNA diluted in total human RNA (HEK 293T cells) was used as a target. Fluorescence was measured with qPCR instrument and normalized to the no target control (NTC, HEK 293T RNA only, dashed line). In each assay, means (n=3) were compared with one-way ANOVA. Pairwise comparisons between target RNA dilutions and NTC were performed using post hoc Dunnett’s test. Data are shown as mean ± SD. *p < 0.05; **p < 0.005; ***p<0.001; ****p < 0.0001.

Type III CRISPR RNA-guided complexes (i.e., Csm and Cmr) bind and cleave complementary single-stranded RNA (ssRNA) targets^25^. Complementary RNA is cleaved in six-nucleotide increments by metal-dependent nucleases (Csm3 or Cmr4) that form the oligomeric “backbone” of the complex^26^. Type III complexes release fragments of the cleaved target, which inactivates ATP polymerization by the Cas10 subunit^26^. Previously, we mutated residues in the Csm3 subunit responsible for target RNA cleavage (D34A), purified the RNase-dead complex (TtCsm^Csm3-D34A^), and showed that the mutant complex provides more sensitive detection of viral RNA than the wildtype complex^16^. To further increase the sensitivity, we set out to determine if TtCsm^Csm3-D34A^ could be used to concentrate sequence-specific RNAs. To test this approach, we mixed ^32^P-labeled target or non-target RNAs with TtCsm^Csm3-D34A^, incubated for 20 minutes, and concentrated the His-tagged complex using nickel-derivatized magnetic beads (Fig. 1a, **Supplementary Fig. 1a**). The beads were concentrated using a magnet, and RNAs were extracted from the bound and unbound fractions. The type III complex captured most of the radiolabeled target RNA (76±5.8%), while non-target RNA primarily remains in the supernatant (Fig. 1b, **Supplementary Fig. 1b, c**). To determine if type III CRISPR-based RNA capture and concentration results in the synthesis of more cyclic nucleotides, we mixed Csm-beads with 120 μL of a sample containing SARS-CoV-2 RNA and total RNA extracted from HEK 293T cells (Fig. 1c, see Methods). After concentrating the beads with a magnet, we resuspended the pellet in a buffer containing α−^32^P-ATP, allowed the cyclic polymerization to proceed, and analyzed the reactions using thin-layer chromatography (TLC). The type III CRISPR-based concentration increases the amount of cA_3_ and cA_4_, as compared to the reaction performed without RNA concentration (Fig. 1c, d, **Supplementary Fig. 1d**).

Previously, we repurposed TtCsm6, a cA_4_-activated ribonuclease, to generate a real-time fluorescent readout for Csm-based RNA detection^16^ (Fig. 1e, **top**). We reasoned that increased cA_4_ levels after RNA enrichment will boost the nuclease activity of TtCsm6 and therefore increase the sensitivity of the RNA detection. To test this hypothesis, we titrated 10^8^ to 10^5^ copies/μL of SARS-CoV-2 N-gene RNA into total RNA extracted from HEK 293T cells, concentrated the target RNA using TtCsm^Csm3-D34A^, resuspended the beads in a buffer containing ATP, and then transferred the polymerization products to a reaction containing TtCsm6 and a fluorescent RNA reporter (i.e., FAM-RNA-Iowa Black FQ). Csm-based RNA enrichment increased the sensitivity of the assay 100-fold compared to the assay without the pull-down (Fig. 1e). Taken together, these results demonstrate how type III-A CRISPR-complexes can be used to capture sequence-specify RNAs, resulting in a higher concentration of cyclic nucleotides, which improves the sensitivity of sequence-specific RNA detection.

### CARF-nucleases Can1 and Can2 exhibit cA_3_- and cA_4_-specific nuclease activities

Csm6 proteins contain an amino-terminal CARF (CRISPR-associated Rossman Fold) and a carboxy-terminal HEPN (Higher Eukaryotes and Prokaryotes Nucleotide-binding) domains^10,12^. Csm6 family proteins form homodimers, and the two CARF-domains bind cA_4_^23,27^ or cA_6_^22^, which activate the C-terminal HEPN nuclease domain. However, the CARF domain of some Csm6 proteins also degrades the cyclic nucleotide, which inactivates the nuclease and may limit the sensitivity of Csm6-based assays^28^. To improve the sensitivity, we sought to identify and incorporate a CARF-nuclease that is activated by but does not degrade cA_4_.

**Fig. 2:**
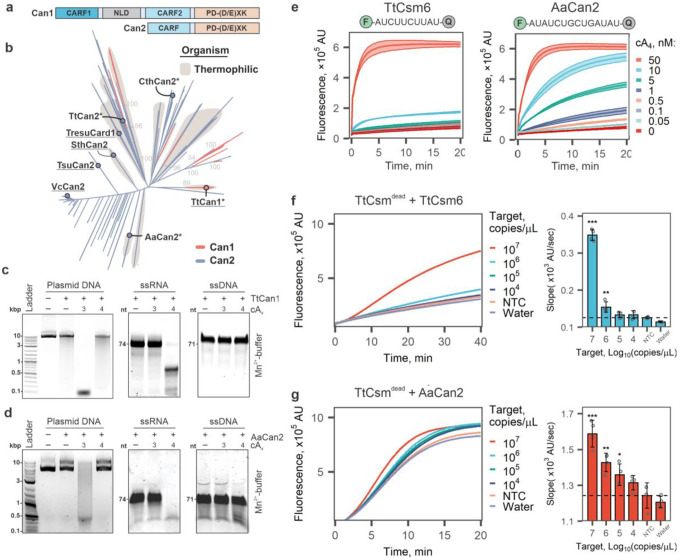
Can1 and Can2 ancillary nucleases cleave RNA or DNA in an activator-dependent manner. **a** Domain organization of Can1 and Can2 proteins. Can2 proteins have two domains – CARF and PD-(D/E)XK superfamily nuclease domain. Can1 is predicted to be derived from Can2 by gene duplication^30^. NLD – nuclease-like domain. **b** Maximum-likelihood phylogeny of 204 Can1 (CARF2 and PD-(D/E)XK nuclease domain) and 3,121 Can2 proteins. Previously studied effectors are underlined on the tree. *, effectors chosen for purification and *in vitro* experiments. **c** Plasmid (15 nM), ssRNA (425 nM), and ssDNA (425 nM) cleavage assay with TtCan1 (200 nM) in the presence of cA_3_ or cA_4_ (20 nM). The reactions were incubated 15 min at 60°C. **d** Cleavage assays with AaCan2 (200 nM) in in the presence cA_4_ or cA_3_ (20 nM). Assays were performed with 15 nM plasmid DNA (left), 425 nM ssRNA or ssDNA (right) for 15 min at 55°C. **e** TtCsm6 (300 nM) and AaCan2 (300 nM) cleavage assays with fluorescent ssRNA reporter (top) in the presence of varying cA_4_ activator concentrations (shown with colors). Data is shown as the mean (center line) of three replicates ± S.D. (ribbon). The optimal fluorescent reporter (top) was determined using RNA library screen in Supplementary Fig. 4. f,g TtCsm RNA detection assays coupled with TtCsm6- **(f)** and AaCan2-based **(g)** readouts were performed using samples with target RNA concentrations ranging from 10^7^ to 10^2^ copies/μL. Samples were prepared by spiking IVT fragments of SARS-CoV-2 N gene into total RNA extracted from nasopharyngeal swab patient sample negative for SARS-CoV-2. Cleavage of fluorescent RNA reporter was detected by measuring fluorescence every 10 sec in a real-time PCR instrument (left). Data were plotted as mean of 4 replicates. Simple linear regression was used to calculate slopes for linear regions of the curves. Bars show mean values (n = 4) ± S.E.M. (right). Data was analyzed with one-way ANOVA followed by multiple comparisons to NTC sample using one-tailed post-hoc Dunnett’s test. *** p < 0.001; ** p < 0.01; * p < 0.05.

CRISPR ancillary nucleases (Can) are another family of recently identified proteins that are activated by cyclic oligoadenylates and lack ring nuclease activity^29–31^. Like Csm6 proteins, Can proteins also contain amino-terminal CARF domains, but the carboxy-terminal nucleases are distinct. The Can1 protein from *Thermus thermophilus* (TtCan1) has a unique monomeric architecture with two non-identical CARF domains, one nuclease-like domain (NLD) and one restriction endonuclease domain (PD-(D/E)XK)^31^, while Can2 nucleases contain a single CARF domain and form symmetrical homodimers^29,30^ (Fig 2a).

To identify Can1 and Can2 orthologs compatible with the TtCsm complex, we generated profile Hidden Markov models (HMMs) to query publicly available microbial genomes and metagenomes from NCBI and JGI. This analysis identified 204 Can1 and 3,121 Can2 proteins. Based on this analysis, we selected TtCan1 and three Can2 orthologs from thermophilic organisms for cloning and expression (Fig. 2b). While previous research demonstrated that metal-dependent nicking of supercoiled DNA by TtCan1 is dependent on activation by cA_4_^31^, the impact of other cyclic oligoadenylates on TtCan1 activity has not been reported. We purified TtCan1 and tested nuclease activity against plasmid DNA in the presence of five different cyclic oligoadenylates (cA_2_-cA_6_) **(Supplementary Fig. 2a-c)**. To our surprise, TtCan1 robustly degrades plasmid DNA to ~100 bp fragments in the presence of cA_3_ and Mn^2+^, while cleavage with cA_4_ is comparable to the background activity in the absence of an activator (Fig. 2c, **left; Supplementary Fig. 2d**). To determine if the TtCan1 nuclease has any sequence preference, we deep-sequenced the cleavage fragments, aligned the reads, and identified cut sites. This analysis failed to identify common sequence motifs that define the cleavage site, suggesting that TtCan1 is a non-sequence specific DNase (**Supplementary Fig. 2e**). Based on the unexpected activation of TtCan1 with cA_3_, we tested several other substrates and discovered that TtCan1 is a cA_4_-dependent single-stranded RNase (ssRNA) but does not cleave ssDNA (Fig. 2c, **Supplementary Fig. 2f, g**). Taken together, our *in vitro* assays show that TtCan1 is a non-sequence specific double-stranded DNase when activated with cA_3_ and a single-stranded RNase when activated with cA_4_.

Can2 genes from Clostridium thermobutyricum (CthCan2), Thermus thermophilus (TtCan2), and Archaeoglobi archaeon JdFR-42 (AaCan2) were cloned and expressed in E. coli (Fig. 2b). However, only AaCan2 purified in quantities sufficient for biochemical assays (**Supplementary Fig. 3a, b**). We systematically tested the activities of AaCan2 against different substrates with a range of cyclic oligoadenylates (**Supplementary Fig. 3c, d**). Like TtCan1, AaCan2 is also a Mn^2+^- and cA_3_-dependent dsDNase (Fig. 2d, left gel; **Supplementary Fig. 3c**), or a ssRNase when activated with cA_4_. The ssRNase activity of AaCan2 is supported by either Mn^2+^ or Mg^2+^ (Fig. 2d, **Supplementary Fig. 3d**). Reproducible cleavage of ssDNA is also detectable for AaCan2, but the activity is Mn^2+^-specific, and robust cleavage requires a higher concentration of cA_4_ (i.e., 45 nM) (Fig. 2d, **Supplementary Fig. 3d**). Cleavage of ssDNA produces a discrete band suggesting that the enzyme processes ssDNA to a minimal cleavage product or that the activity is sequence-specific (Fig. 2d; **Supplementary Fig. 3d**). While cA_4_-dependent activities of AaCan2 are consistent with activities previously reported for the Can2 protein from Treponema succinifaciens^29^ (i.e., TresuCard1; Fig. 2b), cA_3_-dependent dsDNA cleavage has not been previously reported. Collectively, our results demonstrate that Can1 and Can2 function as either dsDNA- or ssRNA-specific nucleases, depending on the cyclic nucleotide activator (i.e., cA_3_ or cA_4_).

### Can2 ancillary nuclease provides sensitive Csm-based RNA detection

To determine if incorporating TtCan1 or AaCan2 improves sensitivity of the Csm-based RNA detection assay, we screened a library of synthetic RNA reporters designed to identify sequences that might be preferred by these nucleases (**Supplementary Table 1**, **Supplementary Fig. 4**). Consistent with our gel-based assays, cA_4_-activated AaCan2 cleaves RNA reporters in the presence of either Mg^2+^ or Mn^2+^, but reactions with Mn^2+^ consistently result in higher fluorescent signal (**Supplementary Fig. 4a, b**). While TtCan1 cleaves the same RNA reporters as AaCan2, cleavage by TtCan1 requires higher concentrations of cA_4_ and produces less fluorescent signal (**Supplementary Fig. 5**).

Having established that AaCan2 is more active than TtCan1, we set out to compare AaCan2 to the sensitivity of TtCsm6, which we used previously^16^. This comparison was performed by measuring cA_4_ concentration-dependent activity for AaCan2 and TtCsm6 using the preferred RNA reporter for each of the respective enzymes (**Supplementary Fig. 4**). AaCan2 produces a similar fluorescent signal to TtCsm6 when activated with 20-fold less cA_4_ (0.5 nM versus 10 nM) (Fig. 2e). Moreover, AaCan2 exhibits an incremental decrease in cleavage rates with decreasing cA_4_, while TtCsm6 exhibits a dramatic (non-linear) drop in the activity. The distinction in activity between these enzymes is consistent with the ring-nuclease activity of TtCsm6 rapidly degrading its activator, while AaCan2 binds and preserves the cyclic nucleotide (**Supplementary Fig. 3e**).

Finally, we incorporated AaCan2 into the type III-based detection assay and benchmarked this combination against TtCsm6-based detection (Fig. 2f, g). The TtCsm6-based assay reliably detects 10^6^ copies/μL of target RNA (Fig. 2f), while AaCan2-based reactions are more sensitive (10^5^ copies/μL) (Fig. 2g). While coupling TtCsm-detection to AaCan2 results in significantly higher sensitivity, it also results in higher background, but this background is only evident in the presence of the TtCsm-complex (Fig. 2f, g), whereas AaCan2 alone demonstrates very little non-specific cleavage (Fig. 2e). This disparity suggests that non-sequence specific activation of the Cas10 polymerase may generate low levels of cA_4_, which stably activates AaCan2, whereas the ring-nuclease of TtCsm6 rapidly degrades cA_4_ limiting the background signal. Collectively, these results demonstrate that coupling AaCan2 with TtCsm^Csm3-D34A^ provides more sensitive RNA detection.

### Incorporating cA_3_-dependent nuclease activity does not provide additional sensitivity of RNA detection

**Fig. 3:**
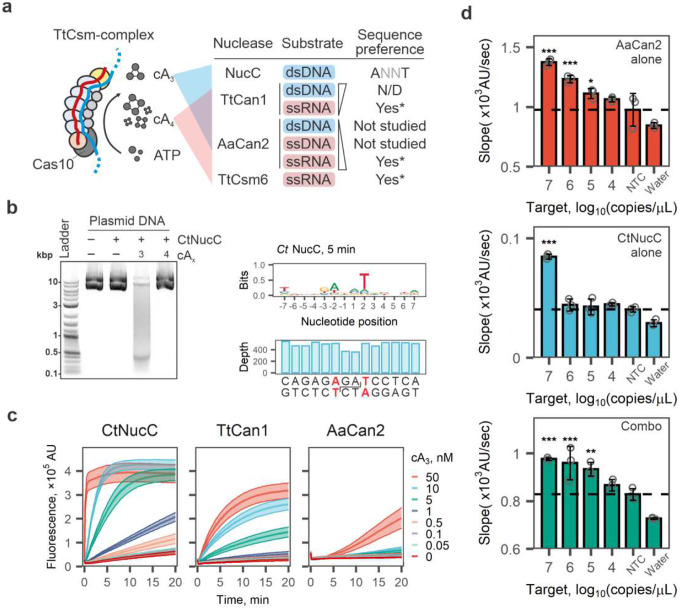
Incorporation of cA_3_-activated nucleases into Csm-based RNA detection assay. **a** The target bound TtCsm complex primarily generates cA_4_ and cA_3_. Schematics summarizes cA_4_- and cA_3_-dependent activities of nucleases biochemically tested. N/D – not detected; Asterisk (*) indicates nucleases that have sequences preferences (Supplementary Fig. 4). **b** *Left panel:* CtNucC (15 nM) is activated by cA_3_ (20 nM) and cleaves plasmid DNA into short fragments in 15 min. *Right panel:* The deep sequencing of DNA fragments generated after 5 min of incubation with CtNucC revealed the preferential cleavage sites (ANNT). The reduced sequencing depth at the cut site is consistent with a cleavage mechanism producing 3’-overhangs that are removed by T4 DNA polymerase when sequencing library is prepared. **c** CtNucC (300 nM), TtCan1 (300 nM) and AaCan2 (300 nM) cleavage assays with fluorescent dsDNA reporter across eight concentrations of cA_3_ (shown with colors). Data is shown as mean (center line) of three replicates ± S.E.M. (ribbon). **d** TtCsm RNA detection assays coupled with AaCan2 (ssRNA reporter), CtNucC (dsDNA reporter) and combination of AaCan2 and CtNucC (both reporters). Reactions were performed using samples with target RNA concentrations ranging from 10^7^ to 10^2^ copies/μL. Samples were prepared by spiking IVT fragment of SARS-CoV-2 N gene in total RNA of SARS-CoV-2 negative nasal swab. Cleavage of the fluorescent reporter was detected by measuring fluorescence every 10 sec in a real-time PCR instrument. Simple linear regression was used to determine slopes for 3 replicates. See Supplementary Fig. 8 for fluorescent curves used in the analysis. Data were plotted as mean (n = 3) ± S.D. and analyzed with one-way ANOVA. All samples were compared to the non-target RNA control (NTC) using one-tailed post-hoc Dunnett’s test. *** p < 0.001; ** p < 0.01; * p < 0.05.

While our assay uses cA_4_-activated collateral cleavage of ssRNA reporters, the TtCsm^Csm3-D34A^-complex also produces cA_3_ (Fig. 1d, **Supplementary Fig. 1d**). We hypothesized that combining cA_3_- and cA_4_-sensing nucleases might enhance the sensitivity of TtCsm-based detection (Fig. 3a). NucC (Nuclease, CD-NTase associated) endonucleases adopt homotrimeric structures forming a 3-fold symmetric pocket for cA_3_ binding^24,32,33^. Binding cA_3_ triggers dimerization of NucC homotrimers juxtaposing pair of active sites to cleave DNA^32,33^. We purified three thermophilic NucC orthologs and tested cA_3_-dependent dsDNA cleavage (**Supplementary Fig. 6**). The NucC from *Clostridium tepidum* (CtNucC) has the highest dsDNase activity and digests plasmid

DNA into 300–400 bp fragments in the presence of cA_3_ (Fig. 3b, **left; Supplementary Fig. 7a**). Deep sequencing of cleavage fragments determined that all purified NucC nucleases have a preference for 5’-ANNT-3’ sequence motif, which is consistent with previously published work^33^ (Fig. 3b, **right; Supplementary Fig. 7b-e**).

Next, we set out to determine if CtNucC and AaCan2 could be combined into a single reaction to improve the sensitivity of RNA detection with TtCsm^Csm3-D34A^. To perform fluorescent assays with CtNucC, we designed a 31-bp dsDNA reporter comprising six repeats of the optimal cleavage site (**Supplementary Table 1**). The lowest concentration of cA_3_ detected by CtNucC is 0.5 nM, which is 10-fold more sensitive than TtCan1 and 100-fold more sensitive than AaCan2 (Fig. 3c). However, TtCsm^Csm3-D34A^ coupled with CtNucC and dsDNA reporter only detects high concentrations of target RNA (i.e., 10^7^ copies/μL; Fig. 3d). Further, combining CtNucC with AaCan2 and matching fluorescent probes (i.e., dsDNA and ssRNA, respectively) (Fig. 3a) into a single reaction does not improve the sensitivity compared to detection with AaCan2 alone (Fig. 3d, **Supplementary Fig. 8a**). While CtNucC is sensitive to cA_3_ activation, the TtCsm-complex may not produce sufficient concentrations of this cyclic nucleotide to increase sensitivity over AaCan2 detection alone.

### Type III CRISPR based RNA capture and detection from patient samples

**Fig. 4:**
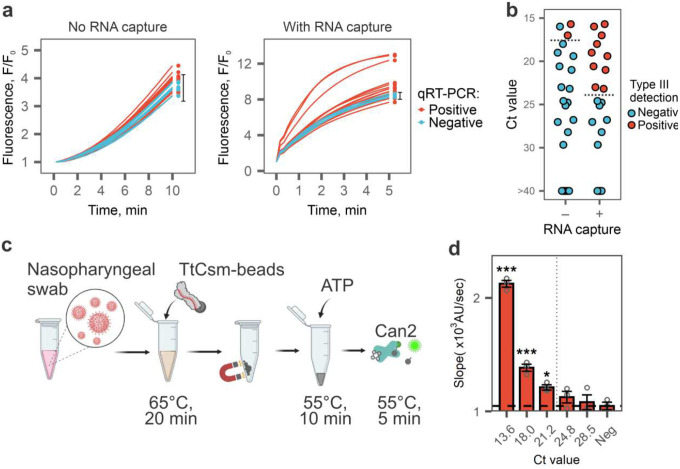
TtCsm-based RNA capture directly detects SARS-CoV-2 in clinical samples. **a** Seventeen SARS-CoV-2 positive (red lines) and six negative (blue lines) RNA samples were tested with TtCsm-AaCan2 detection assay with and without upstream RNA capture. Dots show timepoints that were used to analyze type III detection results. Error bars show mean fluorescence in negative samples (n = 6) ± 2.33 S.D. Reactions that generated signal higher than upper bound of this interval were considered positive for SARS-CoV-2 RNA. **b** Scatter plot showing distribution of Ct values (N1 CDC primers) of RNA samples tested in a. Red dots show samples that tested positive in type III detection, blue shows samples that tested negative. **c** Schematic of TtCsm-based RNA capture assay from nasopharyngeal swab coupled with AaCan2-based fluorescent detection. **d** Nasopharyngeal swab sample positive for SARS-CoV-2 (RT-qPCR Ct = 13.6) was used to make 10-fold serial dilutions in a negative nasopharyngeal swab (Ct > 40). Total of 120 μL of the sample was used for direct detection with TtCsm-based RNA capture assay depicted in c. Bars show mean values (n = 3) ± S.E.M. of the reaction slopes calculated using simple linear regression (Supplementary Fig. 9c). All slopes were compared to the negative control (NTC) with one-way ANOVA and post-hoc one-tailed Dunnett’s test. *** p < 0.001; ** p < 0.01; * p < 0.05.

RNA extracted from nasopharyngeal swabs of COVID-19 patients are complex mixtures of nucleic acids derived from the host, the virus, and microbial communities residing in the upper respiratory tract. To determine if TtCsm complex can capture SARS-CoV-2 RNA in such mixtures, we extracted total RNA from nasopharyngeal swabs of 17 positive and 6 negative patients diagnosed by RT-qPCR (**Supplementary Fig. 9a**). We used 3 μL of each RNA sample to perform the TtCsm-AaCan2 reaction and 120 μL as input for Csm-based RNA capture followed by a polymerization reaction and fluorometric detection with AaCan2. Only samples with the highest viral RNA concentration (Ct <17) tested positive in the TtCsm-AaCan2 reactions. However, adding the Csm-based RNA capture method increases the sensitivity ~100-fold and reliably detects SARS-CoV-2 RNA in patient samples with Ct values ≤23.2, which corresponds to ~10^4^ copies/μL of viral RNA (Fig. 4a, b and **Supplementary Fig. 9b, c**).

RNA extraction kits are expensive, time-consuming, and require specialized equipment. To eliminate this step, we tested if the TtCsm complex can capture and concentrate target RNA directly from a nasopharyngeal swab sample without prior RNA extraction. To identify lysis conditions that do not inhibit activity of the TtCsm-complex, we tested 10 lysis buffer compositions with varying concentrations of detergents (i.e., Triton X-100 or NP-40) and chelators (i.e., EDTA or EGTA) (**Supplementary Fig. 9d**). We mixed Csm-beads with a mock sample made by spiking SARS-CoV-2 RNA fragment into SARS-CoV-2 negative nasopharyngeal swab, added lysis buffer, and incubated for 20 min at 65°C. This heat treatment inactivates SARS-CoV-2, promotes lysis, and allows RNA binding by TtCsm-complex and its downstream activities^34,35^. After pulling down Csm-beads with a magnet, we discarded the supernatant and performed polymerization reactions followed by a TtCsm6-based fluorescent readout. The TtCsm complex detects spiked RNA in the samples treated with Triton X-100 (0.025 – 0.1%) and EGTA (1 mM), while other buffers significantly inhibited Csm-based detection (**Supplementary Fig. 9d**).

Finally, to assess the sensitivity of direct SARS-CoV-2 RNA detection in swab samples using type III capture and AaCan2-based fluorescent detection (Fig. 4c), we used a SARS-CoV-2 positive patient sample (Ct ~13.6) that was 10-fold serially diluted in a negative patient swab sample (Fig. 4d). In this assay, we used lysis buffer supplemented with 0.05% Triton X-100 and 1 mM EGTA. Csm-based RNA capture assay detects SARS-CoV-2 RNA in unprocessed samples (i.e., no RNA purification) with Ct < 21.2 (Fig. 4d, **Supplementary Fig. 9f**), which corresponds to 5×10^4^ copies/μL and ~5-fold less sensitive compared to detection performed using purified RNA (Fig. 4b, **Supplementary Fig. 9e**). To compare the efficiency of direct detection from lysed nasopharyngeal swab relative to detection from extracted RNA, we used three nasopharyngeal swab samples that previously tested positive for SARS-CoV-2 using RT-qPCR (**Supplementary Fig. 9f)**. All three samples tested positive using direct detection from nasal swabs, however direct detection from patient samples resulted in a higher signal-to-noise ratio. This difference suggests that further optimization of the lysis conditions may lead to higher sensitivity (**Supplementary Fig. 9f)**.

## Discussion

CRISPR-based diagnostics have been progressing at a remarkable pace^5^. Development efforts have primarily focused on type V (Cas12) and type VI (Cas13) CRISPR-systems, and the sensitivity of these techniques have improved from picomolar^36^ to attomolar concentrations^28^. However, most CRISPR-based viral diagnostics described to date still require nucleic acid extraction and pre-amplification to reach clinically relevant sensitivities^4^.

In 2021, the first attempts to repurpose type III CRISPR systems for SARS-CoV-2 diagnostics achieved 0.1 – 1 nM sensitivity of RNA detection without pre-amplification^16,17^. More recent improvements using different type III complexes or different ancillary nucleases have been used to detect SARS-CoV-2 RNA in purified RNA samples with ~2–4 fM sensitivity^18,24^. Here, we contribute to the ongoing development of type III systems by developing methods for sequence-specific capture and concentration of target RNAs directly from unprocessed patient samples. This approach enables direct detection of 5×10^4^ copies of SARS-VoV-2 RNA per μL (~90 fM) in clinical samples without laboratory-based RNA extraction or pre-amplification. While the sensitivity of the approach is still inferior to RT-qPCR, it is sufficient to identify infected individuals capable of spreading SARS-CoV-2^37^ and is comparable to rapid antigen tests^2^.

Like Cas13, type III systems also recognize RNA, and the most sensitive detection methods developed to date for either approach rely on collateral nuclease activity to release a fluorescent signal^4^. While Cas13-based methods are currently more sensitive (~50 aM), the intrinsic amplification of RNA recognition by type III system may ultimately improve sensitivity. Type III systems uniquely amplify RNA recognition in two sequential steps: first, through Cas10-mediated polymerization of cOAs and second, through cOA-mediated activation of multi-turnover effectors (e.g., Csm6). In addition to the advantages that might come from consecutive stages of signal amplification, the separation of target recognition by the type III surveillance complex (i.e., Csm or Cmr) from collateral cleavage by ancillary effectors also enables programmable RNA capture. Unlike Cas13, which relies on the same active site for target and non-target collateral cleavage^38^, the RNase-dead TtCsm complex (TtCsm^Csm3-D33A^) can be used to capture and maintain target RNA from a larger volume and concentrate these RNAs for various downstream applications. Incorporating RNA capture increases the sensitivity of type III CRISPR-based diagnostic and allows direct detection in clinical samples without RNA extraction, a prerequisite for most current platforms. We anticipate that further incorporation of type III-based RNA pull-down techniques to bypass RNA extraction, optimization of lysis conditions, and next generation of readouts (e.g., real-time sequencing, digital enzymology, amperometry, etc.) will further boost the sensitivity and minimize time-to-result, bringing type III CRISPR diagnostic to current standards of rapid molecular testing.

Our work to improve type III diagnostics has also uncovered ancillary nuclease activities that are valuable for understanding the basic biology and augmenting applications for biotechnology. Both cA_3_ and cA_4_, but none of the other tested cyclic oligoadenylates (i.e., cA_2_, cA_5_, cA_6_), activate TtCan1 and AaCan2 to cleave specific substrates. TtCan1 is primarily a cA_3_-dependent dsDNase, while AaCan2 is a cA_4_-dependent ssRNase. Can1 nucleases may have emerged from duplication and fusion of ancestral Can2 genes^30,31^, and we hypothesize that this fusion may enable the evolution of mechanisms for recognizing diverse (e.g., non-symmetrical) ligands that activate the effector. Similarly, SAVED (SMODS-Associated and fused to Various Effector Domains) domains appear to be derived from the fusion of two ancient CARF-like domains and are activated by cyclic trinucleotides^39^.

Target RNA binding by type III Csm- or Cmr-complexes triggers synthesis of several cyclic oligoadenylate species in varying ratios^19,24^. We showed that the TtCsm complex predominantly generates cA_4_, while cA_3_ is produced at a lower level. We hypothesize that cOA ratios generated by type III complexes have evolved as a fine-tuned immunomodulatory mechanism that regulates ancillary nuclease activities and infection outcomes. In fact, the genome of *T. thermophilus* (HB8 and HB27 strains) encodes both a cA_4_-activated Csm6 RNase and Can1 CARF-nuclease^31^ that is activated by cA_4_ (RNase) and cA_3_ (DNase). cA_4_ is the primary signal generated by target-bound TtCsm, and RNA cleavage by cA_4_-activated Csm6 nucleases results in growth arrest and facilitates clearance of invading genetic parasites^15^. However, failure to clear the infection through cA_4_-dependent RNase activity by Csm6 would result in continuous polymerization by Cas10 and accumulation of cA_3_, which will activate the TtCan1 DNase. The lack of sequence preference suggests that TtCan1 might degrade the host genome and induce abortive infection and cell death. More work is necessary to understand the diversity of nucleoside-based signal generators and the diversity of signal integrators.

## Methods

### Human clinical sample collection and preparation

Clinical samples were obtained with local IRB approval (protocol #DB033020) and informed consent from patients undergoing testing for SARS-CoV-2 at Bozeman Health Deaconess Hospital. Nasopharyngeal swabs from patients that either tested negative or positive for SARS-CoV-2 were collected in viral transport media. RNA was extracted from all patient samples using the QIAamp Viral RNA Mini Kit (QIAGEN).

### Nucleic acids

Sodium salts of cyclic di-, tri-, tetra-, penta- and hexa-adenosine monophosphates (cA_2_6) were purchased from Biolog Life Science Institute. Fluorescent reporters (RNA and DNA) were purchased from IDT (Supplementary Table 1). The dsDNA reporter was ordered as a duplex from IDT. Target and non-target RNAs of SARS-CoV-2 N-gene were *in vitro* transcribed with MEGAscript T7 (Thermo Fisher Scientific) from PCR products generated from pairs of synthesized overlapping DNA oligos (Supplementary Table 1) (Eurofins). Transcribed RNAs were purified by denaturing PAGE. Total RNA from HEK 293T cells was extracted using TRIzol reagent.

### Non-targeting control (NTC)

Total RNA extracted from SARS-CoV-2 negative nasopharyngeal swabs or total RNA extracted from HEK 293T cells were used as negative controls. RNA extracted from HEK 293T cells was diluted to match the average Ct level (~27) obtained for RNAseP mRNA in RNA samples extracted from nasopharyngeal swabs (Supplementary Table 2). The RT-qPCR for RNase P mRNA was performed using CDC RP primers and probe (2019-nCoV CDC EUA Kit, IDT#10006606).

### Plasmids

Plasmids encoding the type III-A Csm complex frm *Thermus thermophilus* (pCDF-5xT7TtCsm; Addgene #128572 and pACYC-TtCas6–4xcrRNA4.5; Addgene #127764), were a gift from Jennifer Doudna. Vector pCDF-5xT7-TtCsm was used as a template for site-directed mutagenesis to mutate the D33 residue in Csm3 to alanine (D33A) and inactivate Csm3-mediated cleavage of target RNA (pCDF-5xT7-TtCsmCsm3-D34A)^35^. The CRISPR array in pACYC-TtCas6–4xcrRNA4.5 was replaced with a synthetic CRISPR array (GeneArt) containing five repeats and four identical spacers, designed to target the N-gene of SARS-CoV-2 (i.e., pACYC-TtCas6–4xgCoV2N1)^16^. TtCas6 was PCR was PCR-amplified from the pACYC-TtCas6–4xcrRNA4.5 plasmid and cloned between the NcoI and XhoI sites in the pRSF-1b backbone (Millipore Sigma) (pRSF-TtCas6). Expression vector encoding TtCsm6 nuclease, pC0075 TtCsm6 His6-TwinStrep-SUMO-BsaI, was a gift from Feng Zhang (Addgene plasmid #115270)^40^.

Gene fragments encoding for Can1 from *Thermus thermophilus* (TtCan1; NCBI accession=WP_011229147.1), Can2 from *Archaeoglobi archaeon* JdFR-42 (AaCan2; (JGI) IMG gene accession=2730024700), *Clostridium thermobutyricum* (CtCan2; NCBI accession=WP_195972101.1), and *Thermus thermophilus* (TtCan2; NCBI accession=WP_143585921.1), were codon optimized for expression in *E. coli*, synthesized by GenScript, and cloned into pC0075 vector (Addgene #115270) in frame with the N-terminal His6-TwinStrep-SUMO tag using NcoI and XhoI restriction sites to replace the TtCsm6 gene. NucC from *Clostridium tepidum* BSD2780120874b_170522_A10 (CtNucC; NCBI accession= WP_195923598.1), *Elioraea sp*. Yellowstone (EsNucC; NCBI accession= WP_141855040.1) and *Acidimicrobiales bacterium* mtb01 (Amtb01NucC; NCBI accession= TEX45487.1), were cloned into pC0075 backbone using the same restriction sites as for Can1 and Can2 genes.

### Protein expression and purification

Expression and purification of the TtCsm^Csm3-D34A^ complex and TtCsm6 were performed as previously described^16^. TtCan1, AaCan2, CtCan2, TtCan2, CtNucC, EsNucC, and Amtb01NucC) were purified according to the following protocol. Each expression vector was transformed into *Escherichia coli* BL21(DE3) cells and grown in LB Broth (Lennox) (Thermo Fisher Scientific) at 37°C to an OD600 of 0.5. Cultures were then incubated on ice for 1 hour, and then induced with 0.5 mM IPTG for overnight expression at 16°C. Cells were lysed with sonication in Lysis buffer (20 mM Tris-HCl pH 8, 500 mM NaCl, 1 mM TCEP) and lysate was clarified by centrifugation at 10,000 ×g for 25 mins, 4°C. The lysate was heat-treated at 55°C for 45 minutes and clarified by centrifugation at 10,000 *g* for 25 mins at 4°C. His_6_-TwinStrep-tagged protein was bound to a StrepTrap HP column (Cytiva) and washed with Lysis buffer. The protein was eluted with Lysis buffer supplemented with 2.5 mM desthiobiotin and concentrated (10k MWCO Corning Spin-X concentrators) at 4°C. Affinity tags were removed from the protein using His-tagged SUMO protease (100 μL of 2.5 mg/mL protease per 20 mg of protein) during dialysis against SUMO digest buffer (30 mM Tris-HCl pH 8, 500 mM NaCl, 1 mM dithiothreitol (DTT), 0.15% Igepal) at 4°C overnight. The tag and the protease were applied to HisTrap HP column (Cytiva), and the flow-through was concentrated using Corning Spin-X concentrators at 4°C. Finally, the protein was purified using a HiLoad Superdex 200 26/600 size-exclusion column (Cytiva) in storage buffer (20 mM Tris-HCl pH 7.5, 1 mM DTT,400 mM monopotassium glutamate, 5 % glycerol). Fractions containing the target protein were pooled, concentrated, aliquoted, flash-frozen in liquid nitrogen, and stored at −80°C.

### ^32^P-labeling of RNA oligos

Target (SARS-CoV-2 N1) and non-target RNAs were transcribed from PCR extended duplex oligos using home-made T7 RNA polymerase (Supplementary Table 3) (Eurofins). The IVT RNAs were gel purified and dephosphorylated with Quick CIP (NEB) for 20 min at 37°C in 1X CutSmart Buffer (NEB). The phosphatase was inactivated by heating at 80°C for 5 min before 5’ end-labeling the RNAs with T4 polynucleotide kinase (NEB) and [γ−^32^P]-ATP (PerkinElmer) for 30 min at 37°C. The kinase was heat inactivated by heating at 65°C for 20 min.

### Binding and pull-down of RNA oligos with TtCsm

For the experiments shown in Fig. 1b and Supplementary Fig. 1b,c, ^32^P-labeled RNA (25 nM) was incubated with TtCsm^Csm3-D34A^ (160 nM) targeting SARS-CoV-2 N-gene in 1X Binding Buffer (25 mM HEPES, pH 7.5, 150 mM NaCl, 1 mM TCEP) for 20 min at 65°C. The reaction mixtures were added to 10 μL of HisPur Ni-NTA Magnetic beads (ThermoFisher) equilibrated in Binding Buffer and incubated on ice 30 min with vortexing every 10 min. The beads were separated from the supernatant using a magnet and washed with 50 μL 1X binding buffer. The RNA was extracted from supernatant (unbound fraction) and beads (bound fraction) using Acid Phenol: chloroform (Ambion). Extracted RNA was resolved using UREA-PAGE, exposed to a phosphor screen, and imaged on a Typhoon 5 imager (Amersham). Bands corresponding to the IVT RNAs were quantified using ImageJ and the percent bound calculated [bound/(bound + free)*100%].

### Complexing of TtCsm with magnetic beads

The HisPur Ni-NTA Magnetic beads (ThermoFisher) were washed two times with a 1X Binding Buffer (25 mM HEPES, pH 7.5, 150 mM NaCl, 1 mM TCEP). For one reaction, 5 μL of equilibrated beads were mixed with TtCsm^dead^ complex (25 nM) in 1X Binding Buffer (V=50 μL) and incubated for 30 min on ice. The beads with the complex (Csm-beads) were concentrated with a magnet and resuspended in 5 μL of 1× Binding Buffer.

### Thin-layer chromatography (TLC)

For the experiments shown in Fig. 1c, 3 μL of positive sample (target RNA diluted in NTC, 10^10^ copies/μL) or 3 μL of NTC were mixed with TtCsm ^Csm3-D34A^ complex (25 nM) and 250 μM ATP supplemented with [α−^32^P]-ATP (PerkinElmer) in the reaction buffer (20 mM Tris-HCl pH 7.8, 250 mM monopotassium glutamate, 10 mM ammonium sulfate, 1 mM TCEP (tris(2-carboxyethyl)phosphine)), 5 mM magnesium sulfate). The reaction was incubated at 60°C for 1h. For the pull-down reactions, 120 μL of positive or negative samples were mixed with 5 μL of Csm-beads in Binding Buffer (25 mM HEPES, pH 7.5, 150 mM NaCl, 1 mM TCEP) for 10 min at 60°C. The Csm-beads were concentrated with a magnet and the supernatant was discarded. The Csm pellets were resuspended in 30 μL of the reaction buffer and 250 μM ATP supplemented with [α−32P]-ATP (PerkinElmer). Reaction products were phenol-chloroform extracted and resolved on silica TLC plates (Millipore).

Samples (1 μL) were mixed with 100 mM sodium acetate, pH 5.2 (2 μL) and spotted 1.5 cm above the bottom of the TLC plate. The plate was placed inside a 2 L beaker filled to ~0.5 cm with developing solvent (0.2 M ammonium bicarbonate pH 9.3, 70% ethanol and 30% water) and capped with aluminum foil. The plate was run for 2 h at room temperature and dried. TLC plate was exposed to a phosphor screen and imaged with Typhoon phosphor imager. Chemically synthesized standards (2μM) were resolved on the same TLC plate and visualized using UV shadowing.

To test cA_3_ and cA_4_ hydrolysis in the presence of ancillary nuclease, radiolabeled cA_3_ and cA_4_ produced above were mixed with nuclease (500 nM) in the reaction buffer and incubated for 1 hour at 55°C. Reaction products were phenol-chloroform extracted and resolved using thin-layer chromatography (TLC) for 45 min as described above.

### Type III-based RNA detection

3 μL of RNA sample was mixed with 250 μM ATP, 25 nM TtCsm^dead^ complex, 300 nM of nuclease (TtCsm6, AaCan2, or CtNucC) with corresponding reporter in a reaction buffer (20 mM Tris-HCl pH 7.8, 250 mM monopotassium glutamate, 10 mM ammonium sulfate, 1 mM TCEP (tris(2-carboxyethyl)phosphine)), 5 mM magnesium sulfate (for TtCsm6 and CtNucC) or 5 mM manganese(II) chloride (for AaCan2) in a 30 μL reaction. The reporter B8 (300 nM) was used for the reaction with TtCsm6, D7 (300nM) – with AaCan2, and dsDNA probe (300 nM) – with CtNucC. Reactions were incubated at 55°C. Cleavage of fluorescent reporters was detected by measuring fluorescence every 10 sec in a real-time PCR instrument QuantStudio 3 (Applied Biosystems).

### Type III-based RNA pull-down and detection

To bind TtCsm^dead^ complex with the magnetic beads, the HisPur Ni-NTA Magnetic beads (ThermoFisher) were washed two times with a 1X Binding Buffer (25 mM HEPES, pH 7.5, 150 mM NaCl, 1 mM TCEP). For one reaction, 5 μL of equilibrated beads were mixed with TtCsm^dead^ complex (30 nM) in 1X Binding Buffer (V = 50 μL) and incubated for 30 min on ice. The beads with the complex (Csm-beads) were concentrated with a magnet and resuspended in 5 μL of 1× Binding Buffer.

#### Pull-down and detection from RNA sample:

120 μL of sample was mixed with 5 μL of Csm-beads in 1x Binding Buffer for 10 min at 60°C. The Csm-beads were concentrated with a magnet and the supernatant was discarded. The Csm-beads pellet was resuspended in 20 μL of the 1X reaction buffer (20 mM Tris-HCl pH 7.8, 250 mM monopotassium glutamate, 10 mM ammonium sulfate, 1 mM TCEP (tris(2-carboxyethyl)phosphine)), 5 mM magnesium sulfate / manganese(II) chloride) containing ATP (250 μM). The reaction was incubated 10 min at 60°C, the Csm-beads were pelleted, and the supernatant (10μL) was transferred to a new reaction with TtCsm6 (300 nM) and B8 RNA Reporter (300 nM) or AaCan2 (300 nM) and D7 RNA Reporter (300 nM) in 1X reaction buffer (V = 30 μL) (Supplementary Table 1). Reactions were incubated at 55°C. Cleavage of the fluorescent RNA reporter was detected by measuring fluorescence every 10 sec in a real-time PCR instrument QuantStudio 3.

#### Pull-down and detection from nasopharyngeal swab:

120 μL of a nasopharyngeal swab was mixed with 5 μL of Csm-beads in 1X Lysis Buffer and incubated for 20 min at 65°C. Ten lysis buffers compositions were tested. All buffers contained 25 mM HEPES, pH 7.5, 150 mM NaCl, 1 mM TCEP and were supplemented with (A) 0.025% Triton X-100, (B) 0.025% Triton X-100 and 1 mM EDTA, (C1) 0.025% Triton X-100 and 1 mM EGTA, (C2) 0.05% Triton X-100 and 1 mM EGTA, (C3) 0.1% Triton X-100 and 1 mM EGTA, (I) 0.025% NP-40, (J) 0.025% NP-40 and 1 mM EDTA, (K1) 0.025% NP-40 and 1 mM EGTA, (K2) 0.05% NP-40 and 1 mM EGTA, or (K3) 0.1% NP-40 and 1 mM EGTA. Each of the supplements are lettered according to the results presented in Supplementary Fig. 9d. The Csm-beads were concentrated with a magnet and the supernatant was discarded. The Csm-beads pellet was resuspend in 20 μL of the 1x reaction buffer (20 mM Tris-HCl pH 7.8, 250 mM monopotassium glutamate, 10 mM ammonium sulfate, 1 mM TCEP (tris(2-carboxyethyl)phosphine)), 5 mM magnesium sulfate or manganese(II) chloride) containing ATP (250 μM). The reaction was incubated 10 min at 65°C, the Csm-beads were pelleted, and the supernatant (10 μL) was transferred to a new reaction with TtCsm6 (300 nM) and B8 RNA Reporter (300 nM) or AaCan2 (300 nM) and D7 RNA Reporter (300 nM) in 1 × reaction buffer (the final volume of a reaction 30 μL). Reactions were incubated at 55°C. Cleavage of fluorescent RNA reporter was detected by measuring fluorescence every 10 sec in a real-time PCR instrument QuantStudio 3.

### RT-qPCR

RT-qPCR was performed using N1 and RP CDC primers (2019-nCoV CDC EUA Kit, IDT#10006606). RNA was extracted from patient samples with QIAamp Viral RNA Mini Kit (QIAGEN, # 52906) and used for one-step RT-qPCR in ABI 7500 Fast Real-Time PCR System according to CDC protocols (https://www.fda.gov/media/134922/download). In brief, 20 μL reaction included 8.5 μL of Nuclease-free Water, 1.5 μL of Primer and Probe mix (IDT, 10006713), 5 μL of TaqPath 1-Step RT-qPCR Master Mix (ThermoFisher, A15299) and 5 μL of the RNA. Nuclease-free water was used as negative template control (NTC). Amplification was performed as follows: 25°C for 2 min, 50°C for 15 min, 95°C for 2 min followed by 45 cycles of 95°C for 3 s and 55°C for 30 s. To quantify viral RNA in the samples, standard curve for N1 primers was generated using a dilution series of a SARS-CoV-2 synthetic RNA fragment (RTGM 10169, NIST) spanning N gene with concentrations ranging from 10 to 10^6^ copies per μL. Three technical replicates were performed at each dilution. The NTC showed no amplification throughout the 45 cycles of qPCR.

### Nanopore sequencing of DNA cleavage fragments

DNA cleavage fragments were sequenced using Oxford Nanopore with Ligation Sequencing Kit (SQK-LSK109). After incubation with TtCan1 or NucC nucleases, cleavage fragments were column-purified using DNA Clean & Concentrator-5 kit (Zymo Research, D4004) as instructed. Next, for each sample 50 ng of purified DNA was used to prepare sequencing libraries with NEBNext® Ultra^™^ II DNA Library Prep Kit (NEB, E7645S). Briefly, DNA was end-repaired with NEBNext Ultra II End Prep Enzyme Mix, which fills 5’- and removes 3’- overhangs. Next, end-repaired fragments were barcoded with Native Barcoding Expansion kit (ONT, EXP-NBD104) using Ultra II Ligation Master Mix (NEB). Barcoded DNA fragments were pooled together and purified with magnetic beads (Omega Bio-tek, M1378–01). Freshly mixed 80% ethanol was used to wash magnetic bead pellet. Sequencing adapters (AMII) were ligated to barcoded DNA using NEBNext® Quick Ligation Module (NEB, E6056S). Ligation reactions were purified with magnetic beads. SFB buffer (ONT, EXP-SFB001) was used for washes. Resulting DNA library was eluted from the beads in 20 μL of EB buffer (QIAGEN, #19086). DNA concentration was measured with Qubit dsDNA HS Assay (ThermoFisher, Q32851), and 20 g was loaded on the Nanopore MinION (R9.4.1 flow cell). The flow cell was primed, and library was loaded according to Oxford Nanopore protocol (SQK-LSK109 kit). The sequencing run was performed in the high-accuracy base calling mode in the MinKNOW software.

### Sequencing data analysis

Sequenced reads were demultiplexed using guppy-barcoder (ONT) and aligned with minimap2 v2.17-r954-dirty (ax map-ont mode) to the reference plasmid sequence that was modified by adding 1000 bp overlaps at the 5’- and 3’- ends. Overlapping regions were introduced to account for circular nature of the plasmid. Resulting alignments (BAM files) were sorted and indexed using samtools v1.13. Next, *bamtobed* function in bedtools package was used to generate BED files and read coordinates were extracted. Read end coordinates were used to calculate cleavage fragment length distributions and map frequencies of cuts at specific locations (Supplementary Fig. 7). To analyze the sequence preferences of each nuclease, 14 bp windows surrounding read ends were extracted with *getfasta* function from bedtools package. Resulting fasta files were used to calculate position weigh matrices (PWMs) with *getPwmFromFastaFile()* function in DiffLogo R package. Finally, PWMs were plotted as sequence logos using ggseqlogo R package. Sequencing depth around the most frequent cut site for each nuclease was calculated with samtools *depth* function and plotted with ggplot2 package in RStudio.

### RNA reporter’s library

To determine the optimal RNA reporter for each cOA-activated nuclease, we constructed a library of variable RNA sequences tethering a FAM fluorophore to an Iowa Black quencher. These reporters were designed as single-stranded RNA molecules (i.e., 5’FAM-AUNNNNNNAU-IABkFQ-3’; variable region underlined) or to produce a structured RNA (e.g., 5’-FAM-CGCGNNNNNNCGCG-IABkFQ-3’; variable region underlined). The Biostrings package in R was used to construct a library of reporter sequences containing each of the 64 unique trinucleotide combinations possible. Since multiple unique trinucleotides could be included in a single reporter (e.g. 5’-FAM-AUAGAAGAAU-IABkFQ-3’ contains AGA, GAA and AAG), we narrowed our initial library of 64 reporters to remove redundant sequences. This resulted in a library of 24 unique reporter sequences, each of which were integrated into both a single-stranded RNA reporter and a structured RNA reporter (Supplementary Table 1). The R-script used to design these reporters is accessible on GitHub (WiedenheftLab/RNA_reporter_design).

### *In vitro* DNA and RNA cleavage assays

All reactions were performed in a buffer containing 20 mM Tris-HCl pH 7.8, 250 mM monopotassium glutamate, 10 mM ammonium sulfate, 1 mM TCEP, 5 mM magnesium sulfate or 5 mM manganese chloride. Plasmid DNA cleavage assays were performed by incubating 1 μg of Lenti-luciferase-P2A-Neo (Addgene #105621) plasmid with TtCan1, AaCan2 or CtNucC (15–200 nM) in the presence of cOAx (15–45 nM) in 10 μL reaction. After 5–15 min incubation at 60°C for TtCan1 and 55°C for both AaCan2 and CtNucC, Gel Loading Dye, Purple (6X) (NEB) was added and 4 μL was loaded on 1% agarose gel. For ssDNA and ssRNA cleavage assays, 0.425 μM of 71 nt DNA oligo (CGTCGTACCGGTTAGAGGATGGTGCAAGCGTAATCTGGAACATCGTATGGGTATGCCCACGGTGTCCACGGCG, Eurofins) or 0.425 μM of 74 nt IVT RNA SARS-CoV-2 N-gene (Supplementary Table 3) were incubated with TtCan1 (200 nM) or AaCan2 (200 nM) in the presence of cOAx (20–45 nM) in 10 μL. After 5–15 min incubation at 60°C for TtCan1 and at 55°C for AaCan2, 2X RNA Loading Dye (NEB) was added and 10 μL was loaded on 12% UREA PAGE.

### Phylogenetic analysis of Can1 and Can2 proteins

A DELTA-BLAST was initiated, using previously described Can1 and Can2 proteins as queries^29–31^ to generate individual lists of closely related proteins with an e-value cutoff of 10^−4^ and 50% query coverage. The resulting sequences were then used as queries to initiate a PSI-BLAST search with an E-value cutoff of 10^−4^ and 50% query coverage. This step was repeated until convergence and redundant sequences were removed with CD-HIT v4.7^41^. In case of Can1, sequences from a previously published dataset^14^ that contain two CARF domains and a nuclease domain were used to generate multiple sequence alignment of Can1-related proteins. In total, 29 sequences of Can1-related proteins and 2,531 sequences of Can2-related proteins were used separately to generate multiple sequence alignment with a local version of MAFFT v7.429^42^ (--localpair --maxiterate 1000). The generated alignments for Can1 and Can2 were curated with MaxAlign v1.1^43^ to remove misaligned or non-homologous sequences. The resulting dataset—comprised of 29 Can1-like and 1,283 Can2-like proteins, respectively—were then individually realigned with MAFFT and HMMbuild^44^ (HMMER v3.2.1) was used to generate HMM profiles from each alignment. The resulting profiles were used to search a local database of prokaryotic genomes from NCBI (downloaded on June 11, 2021) and list of sequences identified in BLAST search from previous steps. An initial search performed with these HMM profiles identified 1,442 Can1 and 5,431 Can2 homologs, which were manually filtered according to the presence of domains that define each protein, as well as the presence of conserved residues found in CARF and nuclease domains. The resulting set of 204 Can1 and 3,121 Can2 proteins were merged into a single file and aligned in MAFFT (LINSI option) for downstream phylogenetic analyses. Next, Trimal v1.4^45^ was used to remove columns in the alignment comprised of ≥70% gaps. Thermostable homologs of Can1 and Can2 were annotated according to organisms that they are originated. ProtTest v3.4.2^46^ was used to select an evolutionary model, and a phylogenetic tree was constructed in IQ-TREE v1.6.1^47^ using the recommended model (i.e., LG+G+F). The phylogenetic tree was plotted using the ggTree package in R^48^.

### Phylogenetic analysis of NucC

A phylogenetic tree of NucC proteins was generated using the same methods as described above for Can1/Can2 proteins. Briefly, DELTA-BLAST and PSI-BLAST searches with previously identified NucC proteins^32^ generated a list of closely related proteins (e-value cutoff of 10^−4^ and minimum 50% query coverage). The resulting dataset was filtered with CD-HIT v4.7 to remove redundant sequences. The resulting 1,230 NucC sequences were aligned with MAFFT (--localpair --maxiterate 1000), and poorly aligned and highly gapped sequences were removed with MaxAlign. The resulting set of 896 NucC sequences were re-aligned with MAFFT as previously described, and the resulting alignment was used to generate a NucC HMM profile which we used to search within prokaryotic genomes from NCBI. This search identified 1,774 hits, which were filtered according to the presence of restriction endonuclease-like domain (i.e., ID_x30_EAK-motif containing), gate-loop and cA_3_ binding domains and were aligned with MAFFT. The remaining NucC homologs were curated according to organisms they are originated from to identify thermostable NucC homologs. The resulting alignment of 1,510 NucC proteins with 21 thermostable homologs was used to generate a phylogenetic tree with FastTree v2.1.10^49^ and was plotted using the ggTree package in R.

### QUANTIFICATION AND STATISTICAL ANALYSIS

All statistical analyses were performed in RStudio. Analysis of Variance Models (ANOVA) were calculated with *aov()* function in the stats R package. Multiple comparisons between positive samples and negative controls were performed using Dunnett’s test with multcomp R package. Reaction slopes were determined by extracting coefficients from linear models fitted to fluorescence data with *lm()* function in R. The linear regions of the fluorescence curves were identified using rolling regression with *auto_rate()* function in respR package. Patient samples (n = 17) for viral detection assays were randomly selected from a sample database (n = 858) with base R function *sample()*. Statistical threshold for detecting SARS-CoV-2 in patient samples with Csm-based assay was set as mean of negative control ± 2.33 S.D., which captures 98% of variation in negative samples (2% false positive). Samples with z-score > 2.33 were considered positive for SARS-CoV-2. Z-scores were calculated in R using following formula: Z = (F_sample_ - μ_neg_)/σ_neg_, where F_sample_ is fluorescence measured in a sample, μ_neg_ is mean of the negative control, σ_neg_ is standard deviation of the negative control. Statistical significance levels used in the figures are *** p < 0.001, ** p < 0.01, and * p < 0.05.
